# An Option of Conservative Management of a Duodenal Injury Following Laparoscopic Cholecystectomy

**DOI:** 10.1155/2014/398545

**Published:** 2014-10-21

**Authors:** MA Modi, SS Deolekar, AK Gvalani

**Affiliations:** Department of General Surgery, King Edward Memorial Hospital and Seth Gordhandas Sunderdas Medical College, Parel, Mumbai 400012, India

## Abstract

Duodenal injury following laparoscopic cholecystectomy is rare complications with catastrophic sequelae. Most injuries are attributed to thermal burns with electrocautery following adhesiolysis and have a delayed presentation requiring surgical intervention. We present a case of a 47-year-old gentleman operated on for laparoscopic cholecystectomy with a bilious drain postoperatively; for which an ERC was done showing choledocholithiasis with cystic duct stump blow-out and a drain in the duodenum suggestive of an iatrogenic duodenal injury. He was managed conservatively like a duodenal fistula and recovered without undergoing any intervention.

## 1. Introduction

Bowel injuries are an uncommon complication after laparoscopic cholecystectomy but are an extremely serious complication when they do occur. Although the reported incidence rate of bowel injury is between 0.05% and 0.14% [1, 2], a large number of cases do not get reported. Most of these cases are due to injury caused while thermal injury and insertion of trocar and rarely due to dissection or adhesiolysis [3]. Most of these duodenal injuries are managed surgically, either laparoscopic or laparotomy. We present a case of an iatrogenic duodenal injury postlaparoscopic cholecystectomy managed conservatively.

## 2. Case Presentation

**Figure 1:**
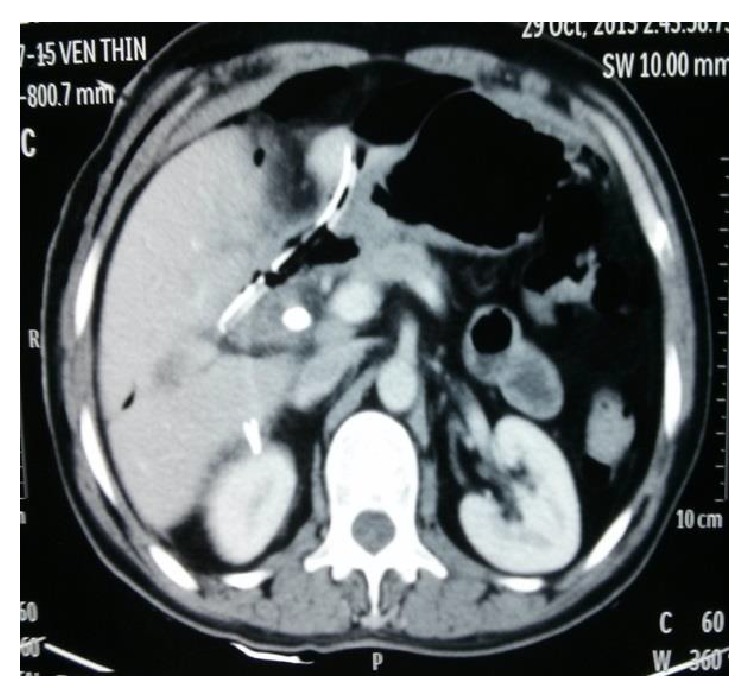
CT scan showing the drain in the 2nd part of duodenum.

**Figure 2:**
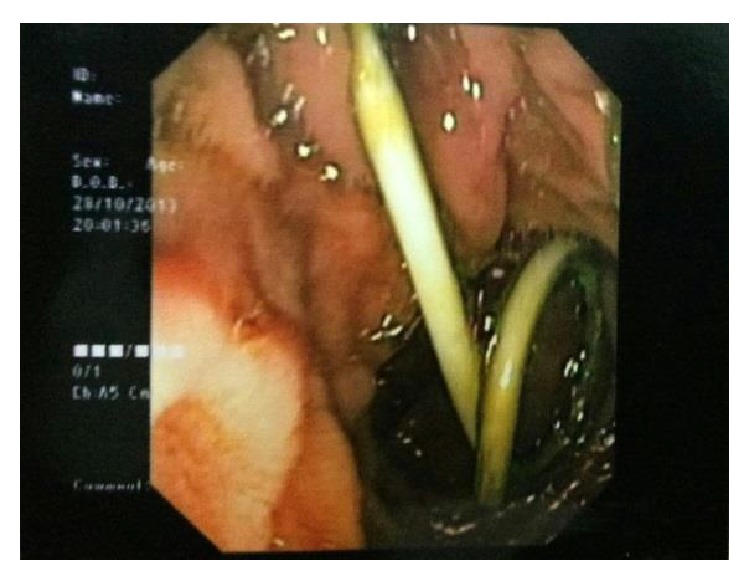
Postoperative ERCP showing a coiled drain in the duodenum.

**Figure 3:**
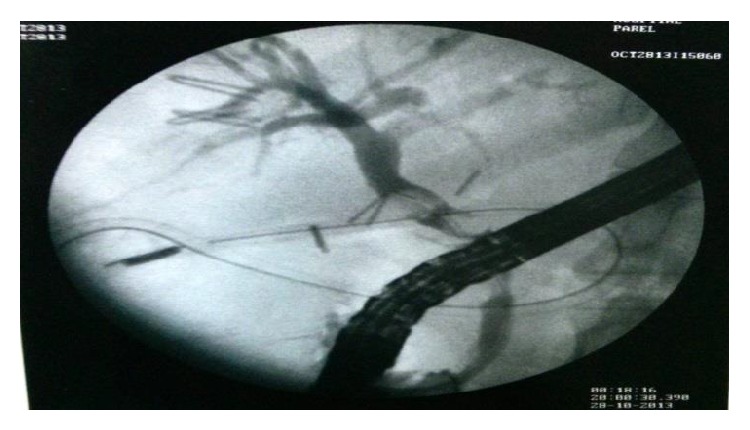
Postoperative ERCP showing a mid-CBD calculi and cystic duct stump blow-out. A drain (ryles tube) is seen in the region of duodenum.

**Figure 4:**
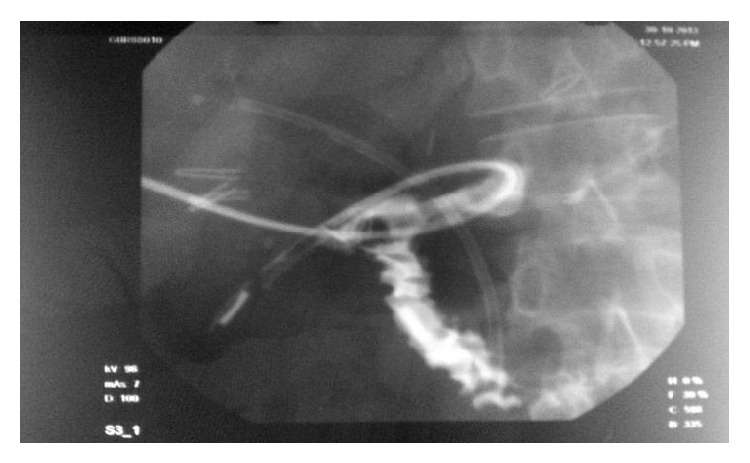
Tube conray gram showing the dye filling up in the duodenum without evidence of any intraperitoneal leak.

**Figure 5:**
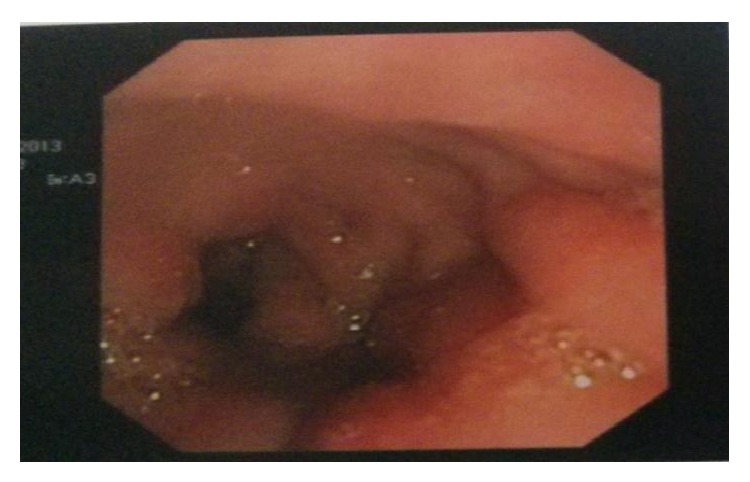
Duodenum at stent removal after removal of drain.

A 47-year-old gentleman had a 10-day history of painful obstructive jaundice. Ultrasonography revealed chronic cholecystitis with choledocholithiasis. EUS revealed a slightly dilated common bile duct (CBD) 9 mm with multiple stones impacted just above the ampulla in the lower CBD with multiple gall stones. ERC with sphincterotomy, stone extraction, and stent placement was done; complete clearance was achieved. A month later, he underwent laparoscopic cholecystectomy at a community hospital; intraoperatively he had dense omental adhesions around Calot's triangle which were separated and the wide cystic duct was identified and clipped. In view of bleeding, while adhesiolysis a drain was placed postoperatively. On the second postoperative day, the drain was bilious in nature and was referred to our centre for further management. On arrival he was vitally stable with minimal epigastric tenderness and had a bilious drain. A CT scan done revealed a drain in the second part of the duodenum (Figure 1). We sent the patient for ERC in view of suspected duodenal and biliary injury (Figure 2). Duodenoscopy revealed the tip of the drain at the junction of D1-D2, previous stent was removed, and cholangiogram revealed mid-CBD calculi with cystic duct stump blow-out (Figure 3). Stone extraction was done and another stent was placed. The patient did not show any signs of sepsis; hence we managed him conservatively with the drain behaving like a tube duodenostomy with a daily output of around 200 mL. He was started on orals which he tolerated well. A conray gram done after 3 weeks, through the drain, showed no intraperitoneal leak and free flow of contrast into the duodenum (Figure 4). The drain was clamped and removed and this was followed by CBD stent removal (Figure 5). On 6-month follow-up, he is doing well.

## 3. Discussion

Duodenal injuries are infrequent complications of laparoscopic cholecystectomy mostly seen due to dense adhesions. Acute and chronic inflammation of gallbladder causes dense adhesions around Calot's triangle and duodenal wall, thus rendering laparoscopic dissection more difficult and sometimes unsafe. In these cases, injury to duodenum may be difficult to appreciate, especially in patients in whom the apex of the duodenal bulb is tented up in front of the neck of the gallbladder [4, 5]. The reason for duodenal injury could be ascribed to the use of cautery during dissection. Thermal bowel injuries usually are not seen at the time of laparoscopic procedures and are diagnosed much later, when transmural necrosis progresses to perforation [2, 4, 6, 7]. The time of injury to onset of symptoms can vary from 18 hours to 14 days [7]. Only in 2 published cases of duodenal perforation due to electrocautery burns during LC injury was observed during surgery [6, 8]. In the other cases, focal thermal injury was unrecognized during the procedure and was presented much later until perforation of duodenal wall developed (1st to 16th postoperative day) [9–13]. Drains eroding into bowel are very well known. Trocar insertion is an early cause of duodenal injuries especially in an inflammatory condition. We do not know the exact reason for the duodenal perforation. Trocar related injury, thermal injury, and drain fistulising into the duodenum are possible hypothesis, the latter two theoretically being less likely on postoperative day two.

The late recognition of these injuries in patients leads to peritonitis and sepsis which contributes to the relatively high associated mortality. Deziel et al. [2] reported an 8.3% mortality rate among 12 patients with duodenal injuries in their analysis of 77,604 cases. El-Banna et al. [6] noted that three of four duodenal thermal injuries complicating laparoscopic cholecystectomy were fatal. Huang et al. [1] reported that 4 out of 19 (21.05%) patients with duodenal injury expired in their study of 39,238 LC cases.

Despite reports of the successful laparoscopic repair of duodenal perforation [14] during laparoscopic cholecystectomy discovered during surgery [8] or diagnosed after surgery [15], most authorities managed this dangerous complication by immediate laparotomy to assess the abdomen and secure a safe repair [2, 6, 9, 16–18].

The site of duodenal injury is important in the surgical management and prognosis. Injuries to duodenal bulb or superior flexure (as in our case) have a better prognosis than descending duodenal injuries around the ampulla of Vater which invariably require complex surgical management and have a high mortality rate [19].

Duodenal injury is a rare but a dangerous complication of laparoscopic cholecystectomy and is associated with a high mortality. In most cases, these injuries are attributed to careless adhesiolysis with electrocautery, remain unrecognized during the procedure, and present later as peritonitis. The authors advocate placement of a drain in all patients with dense adhesions and suspicion of injury in cases of difficult laparoscopic cholecystectomy. At the end of the procedure an accurate control of the abdomen is absolutely necessary.

In conclusion, the authors wish to stress that the general practise for duodenal injuries should be to repair either laparoscopically or surgically and nonoperative management of duodenal injury in selective patients with controlled drainage.
